# Supplementary material for: Intrinsically regulated learning is modulated by synaptic dopamine signaling
Source: eLife. 2018 Aug 30;7:e38113. doi: 10.7554/eLife.38113 (PMC6133552; doi:10.7554/eLife.38113)
Supplement: Transparent reporting form [file elife-38113-transrepform.docx]

***eLife’s* transparent reporting form**

We encourage authors to provide detailed information *within their submission* to facilitate the interpretation and replication of experiments. Authors can upload supporting documentation to indicate the use of appropriate reporting guidelines for health-related research (see [EQUATOR Network](http://www.equator-network.org/%20)), life science research (see the [BioSharing Information Resource](https://biosharing.org/)), or the [ARRIVE guidelines](http://www.plosbiology.org/article/info:doi/10.1371/journal.pbio.1000412) for reporting work involving animal research. Where applicable, authors should refer to any relevant reporting standards documents in this form.

If you have any questions, please consult our Journal Policies and/or contact us: [editorial@elifesciences.org](mailto:editorial@elifesciences.org).

**Sample-size estimation**

- You should state whether an appropriate sample size was computed when the study was being designed
- You should state the statistical method of sample size computation and any required assumptions
- If no explicit power analysis was used, you should describe how you decided what sample (replicate) size (number) to use

Please outline where this information can be found within the submission (e.g., sections or figure legends), or explain why this information doesn’t apply to your submission:

The sample size for this experiment was calculated based on previous literature, Cohen's recommendations (Cohen, 1988) and the software G*Power. This information can be found in page 14.

**Replicates**

- You should report how often each experiment was performed
- You should include a definition of biological versus technical replication
- The data obtained should be provided and sufficient information should be provided to indicate the number of independent biological and/or technical replicates
- If you encountered any outliers, you should describe how these were handled
- Criteria for exclusion/inclusion of data should be clearly stated
- High-throughput sequence data should be uploaded before submission, with a private link for reviewers provided (these are available from both GEO and ArrayExpress)

Please outline where this information can be found within the submission (e.g., sections or figure legends), or explain why this information doesn’t apply to your submission:

Performing real biological replicates with higher-order organisms such as humans is difficult (e.g., one does not have a well-defined lineage for humans). Indeed, is impossible to ensure that all our participants were exposed during their lives to the same external factors. In our study, we use the same learning paradigm than that we employed in Exp. 3 of our previous work (Ripollés et al., 2016, *eLife).* We conducted several analyses to show that the behavioural results of the placebo session (for both learning measures and subjective ratings) indeed replicate our previous data. This information can be found on pages 5 to 7.

**Statistical reporting**

- Statistical analysis methods should be described and justified
- Raw data should be presented in figures whenever informative to do so (typically when N per group is less than 10)
- For each experiment, you should identify the statistical tests used, exact values of N, definitions of center, methods of multiple test correction, and dispersion and precision measures (e.g., mean, median, SD, SEM, confidence intervals; and, for the major substantive results, a measure of effect size (e.g., Pearson's r, Cohen's d)
- Report exact p-values wherever possible alongside the summary statistics and 95% confidence intervals. These should be reported for all key questions and not only when the p-value is less than 0.05.

Please outline where this information can be found within the submission (e.g., sections or figure legends), or explain why this information doesn’t apply to your submission:

|  | **Test Used** | **N** | **Pages** |
| --- | --- | --- | --- |
|  |  |  |  |
| *Replication: comparing learning scores between the placebo session and our previous work* | Mixed between-within Repeated Measures ANOVA | 50 | 6, 20 |
| *Replication: comparing subjective ratings between the placebo session and our previous work* | Mixed between-within Repeated Measures ANOVA | 46 | 6,7, 20 |
| *Drug effect for the learning scores of the control condition (M-)* | Paired t-tests | 26 | 8,21 |
| *Drug effect for the subjective ratings of the control condition (M-)* | Paired t-tests | 24 | 8,21 |
| *Drug effect for the learning scores of the main learning condition (M+)* | Paired t-tests, Repeated Measures ANOVA | 26 | 7, 21,31 |
| *Drug effect for the subjective ratings of the main learning condition (M+)* | Paired t-tests, Repeated Measures ANOVA | 26 | 7, 21,32 |
| *Correlations between the learning scores (M- and M- conditions) and the PAS or the drug-dosage* | Spearman´s rho with FDR correction | 24 | 8,9, 22,33 |
| *Group (hedonic +, hedonic -) comparisons for the learning scores of the main condition (M+)* | Mann-Whitney tests, Mixed between-within Repeated Measures ANOVA | 24 | 10, 22,32 |
|  |  |  |  |

(For large datasets, or papers with a very large number of statistical tests, you may upload a single table file with tests, Ns, etc., with reference to sections in the manuscript.)

**Group allocation**

- Indicate how samples were allocated into experimental groups (in the case of clinical studies, please specify allocation to treatment method); if randomization was used, please also state if restricted randomization was applied
- Indicate if masking was used during group allocation, data collection and/or data analysis

Please outline where this information can be found within the submission (e.g., sections or figure legends), or explain why this information doesn’t apply to your submission:

In this work, one group of participants completed three learning sessions after the intake of different drugs. Drugs were administered in a double-blind masked fashion (a capsule containing the treatment) and were randomized and balanced with a within-subjects design. This information is located in pages 13-14 and 35.

**Additional data files (“source data”)**

- We encourage you to upload relevant additional data files, such as numerical data that are represented as a graph in a figure, or as a summary table
- Where provided, these should be in the most useful format, and they can be uploaded as “Source data” files linked to a main figure or table
- Include model definition files including the full list of parameters used
- Include code used for data analysis (e.g., R, MatLab)
- Avoid stating that data files are “available upon request”

Please indicate the figures or tables for which source data files have been provided:

No source data is provided.
